# Alterations in the Processing of Platelet APP (Amyloid Beta Precursor Protein) in Alzheimer Disease: The Possible Nexus

**DOI:** 10.1002/npr2.12525

**Published:** 2025-01-05

**Authors:** Hayder M. Al‐kuraishy, Ghassan M. Sulaiman, Hamdoon A. Mohammed, Retaj A. Dawood, Ali K. Albuhadily, Ali I. Al‐Gareeb, Mosleh M. Abomughaid, Daniel J. Klionsky

**Affiliations:** ^1^ Department of Clinical Pharmacology and Medicine, College of Medicine Mustansiriyah University Baghdad Iraq; ^2^ Division of Biotechnology, Department of Applied Sciences University of Technology Baghdad Iraq; ^3^ Department of Medicinal Chemistry and Pharmacognosy, College of Pharmacy Qassim University Buraydah Qassim Saudi Arabia; ^4^ Department of Biology, College of Science Al‐Mustaqbal University Hillah Iraq; ^5^ Jabir ibn Hayyan Medical University Najaf Iraq; ^6^ Department of Medical Laboratory Sciences, College of Applied Medical Sciences University of Bisha Bisha Saudi Arabia; ^7^ Life Sciences Institute University of Michigan Ann Arbor Michigan USA

**Keywords:** activated platelets, Alzheimer's disease, amyloid precursor protein, pathogenesis

## Abstract

Alzheimer's disease (AD) is the most common neurodegenerative disease associated with the development of dementia. The hallmarks of AD neuropathology are accumulations of amyloid peptide (Aβ) and neurofibrillary tangles (NFTs). Aβ is derived from the processing of APP (amyloid beta precursor protein) by BACE1 (beta‐secretase 1) and γ‐secretase through an amyloidogenic pathway. However, processing of APP by ADAM10/α‐secretase (ADAM metallopeptidase domain 10) enzymes through a non‐amyloidogenic pathway produces soluble APP alpha (sAPPα), which has a neuroprotective effect. It has been shown that activated platelets are implicated in the pathogenesis of AD, which also increases platelet activation. Under physiological conditions, platelets regulate synaptic plasticity and increase neuronal differentiation by regulation of the inflammatory response. However, overactivated platelets contribute to the pathogenesis of AD. Activated platelets represent the main source of circulating APP and Aβ that may be involved in AD neuropathology. Therefore, there is a close relationship between AD neuropathology and activated platelets. This review discusses the potential role of platelets in the pathogenesis of AD, and how targeting of activated platelets may reduce AD neuropathology.

## Abstract 2

This review examines the role of activated platelets in Alzheimer disease (AD) pathology, highlighting their contribution to amyloid peptide (Aβ) accumulation and neurofibrillary tangles. While platelets normally support neuronal health and synaptic plasticity, their overactivation may exacerbate AD. Targeting activated platelets presents a potential strategy to mitigate AD neuropathology.

## Graphical abstract

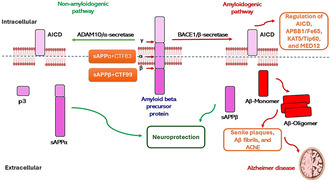

**Abbreviations**
AD: Alzheimer disease
Aβ: amyloid peptide
BBB: blood—brain barrier
MCI: mild cognitive impairment
NFTs: neurofibrillary tangles
PAF: platelet activating factor
sAPPα: soluble APP alpha

## 1 Introduction

**FIGURE 1:**
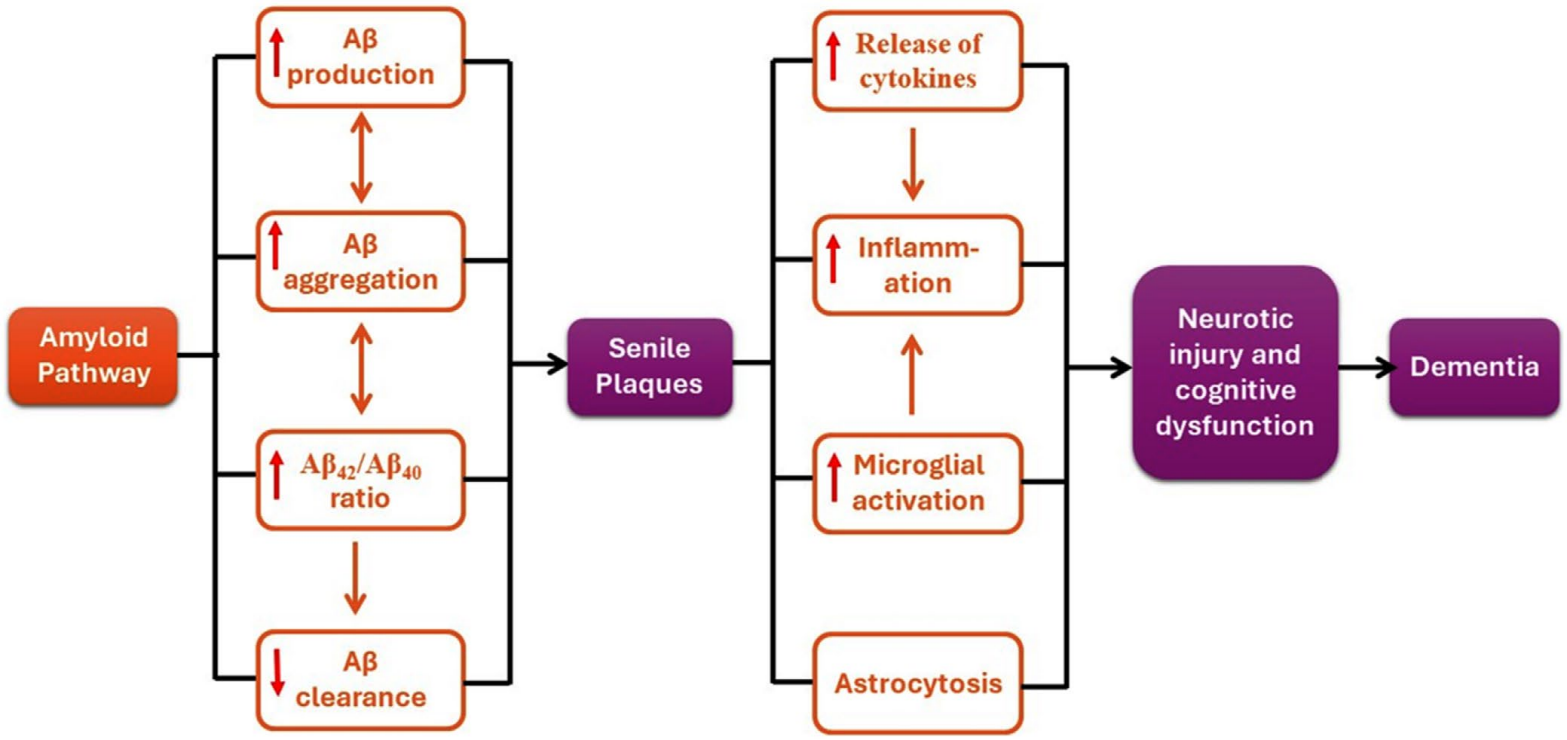
The pathophysiology of AD. APP cleavage by BACE1 generates a 99 amino acid C‐terminal fragment that can be processed by γ‐secretase, ultimately resulting in smaller fragments including Aβ_40_ and Aβ_42_. These two fragments have different conformations such that Aβ_42_ is more likely to form amyloids. Extracellular deposits of Aβ lead to the formation of amyloid beta plaques/senile plaques. These plaques can lead to an immune response, which in turn causes neural injury, ultimately leading to dementia.

Alzheimer disease (AD) is the most common neurodegenerative disease associated with the development of dementia. AD affects 10% of people aged > 65 years, and 50% of people aged > 85 years [1]. Therefore, AD is regarded as an age‐related disease; however, early‐onset AD may start in individuals aged < 65 years [2]. The most common type of AD is sporadic AD, which contributes to 90% of all AD cases [3]. Only 10% of AD, known as familial, is developed due to genetic and inherited factors [4]. The hallmarks of AD neuropathology are extracellular and intracellular accumulations of amyloid peptide (Aβ) and neurofibrillary tangles (NFTs), correspondingly [5, 6]. Aβ is derived from the processing of APP (amyloid beta precursor protein) by BACE1/β‐secretase (beta‐secretase 1) and γ‐secretase through an amyloidogenic pathway [7, 8]. In contrast, processing of APP by ADAM10/α‐secretase (ADAM metallopeptidase domain 10) through a non‐amyloidogenic pathway produces soluble APP alpha (sAPPα), a form of the protein having a neuroprotective effect [9]. Both Aβ and MAPT/tau (microtubule associated protein tau) are present normally in the brain and are involved in neurotransmission release and axonal transport, respectively [10]. However, aggregated Aβ and mutant hyperphosphorylated MAPT/tau induce oxidative stress, neuroinflammation, synaptic injury, and neuronal apoptosis [11] (Figure 1). These neuropathological changes are associated with atrophy of cerebral cortex gray matter, and progressive neuronal loss in the parietal lobes and hippocampus [12].

Of note, AD is considered as a primary brain neurodegenerative disease. However, in late AD there are many systemic manifestations such as fatigue and retarding physical activity due to progressive neurodegeneration [13]. Remarkably, prodromal AD symptoms are initiated a decade prior to cognitive impairment and dementia [14]. Metabolic and biochemical changes observed in the AD brain are also present in the peripheral cells such as skin fibroblasts [15]. In addition, mitochondrial dysfunction in AD is present both in the central nervous system/CNS and peripheral cells such as lymphocytes [16]. Therefore, it has been hypothesized that AD can induce systemic manifestations, and the reverse is also true. For example, systemic inflammatory disorders, obesity, and diabetes increase AD risk [17]. In addition, AD can induce systemic changes through involving the center of the autonomic nervous system with a subsequent reduction of aerobic fitness [18]. These findings suggest that AD is associated with systemic complications.

Moreover, activated platelets are implicated in the pathogenesis of AD, which also increases platelet activation. Oxidative stress in the platelets is augmented in AD patients compared to healthy controls [18]. Under physiological conditions, platelets regulate synaptic plasticity and increase neuronal differentiation by regulation of the inflammatory response [19]; however, overactivated platelets contribute in the pathogenesis of AD [19]. Activated platelets represent the main source of circulating APP and Aβ that may be involved in AD neuropathology [20, 21]. Thus, there is a close relationship between activated platelets and AD neuropathology. Therefore, this review discusses the potential role of platelets in the pathogenesis of AD, and how targeting of activated platelets may decrease AD neuropathology.

## 2 Overview of Platelet Functions

Platelets are circulating cells involved in blood homeostasis and prevention of bleeding after trauma. Platelets are derived from bone marrow megakaryocytes, and they have a short lifespan of approximately 10 days [22]. Platelets have no nuclei, but comprise dense granules, alpha granules, lysosomes, and a canalicular system for storage and processing of precursor peptides [23]. Additionally, platelets have non‐hemostatic functions such as regulation of immunity and inflammation [24]. Platelets, through the expression of TLR (toll like receptor) proteins, can bind and kill many pathogens or provide them to the immune system [25]. The platelets can release many anti‐inflammatory and pro‐inflammatory cytokines, which regulate the recruitment of leukocytes [26]. In addition, platelets express many cytokines and inflammatory molecules which are necessary for leukocyte migration and differentiation. For example, platelets release IL1B/IL‐1β (interleukin 1 beta), PDGF (platelet derived growth factor) and chemokines [27]. As well, platelets affect the adaptive immune response by releasing CD40 molecules [23]. Interestingly, platelets have all of the elements for protein synthesis and for the transcription of non‐coding RNAs and microRNAs [24]. Furthermore, platelets can synthesize and release coagulation factors, adhesive proteins, proteoglycans, protease inhibitors, and immunoglobulin [25, 27]. Therefore, platelets modulate both adaptive and innate immunity.

**FIGURE 2:**
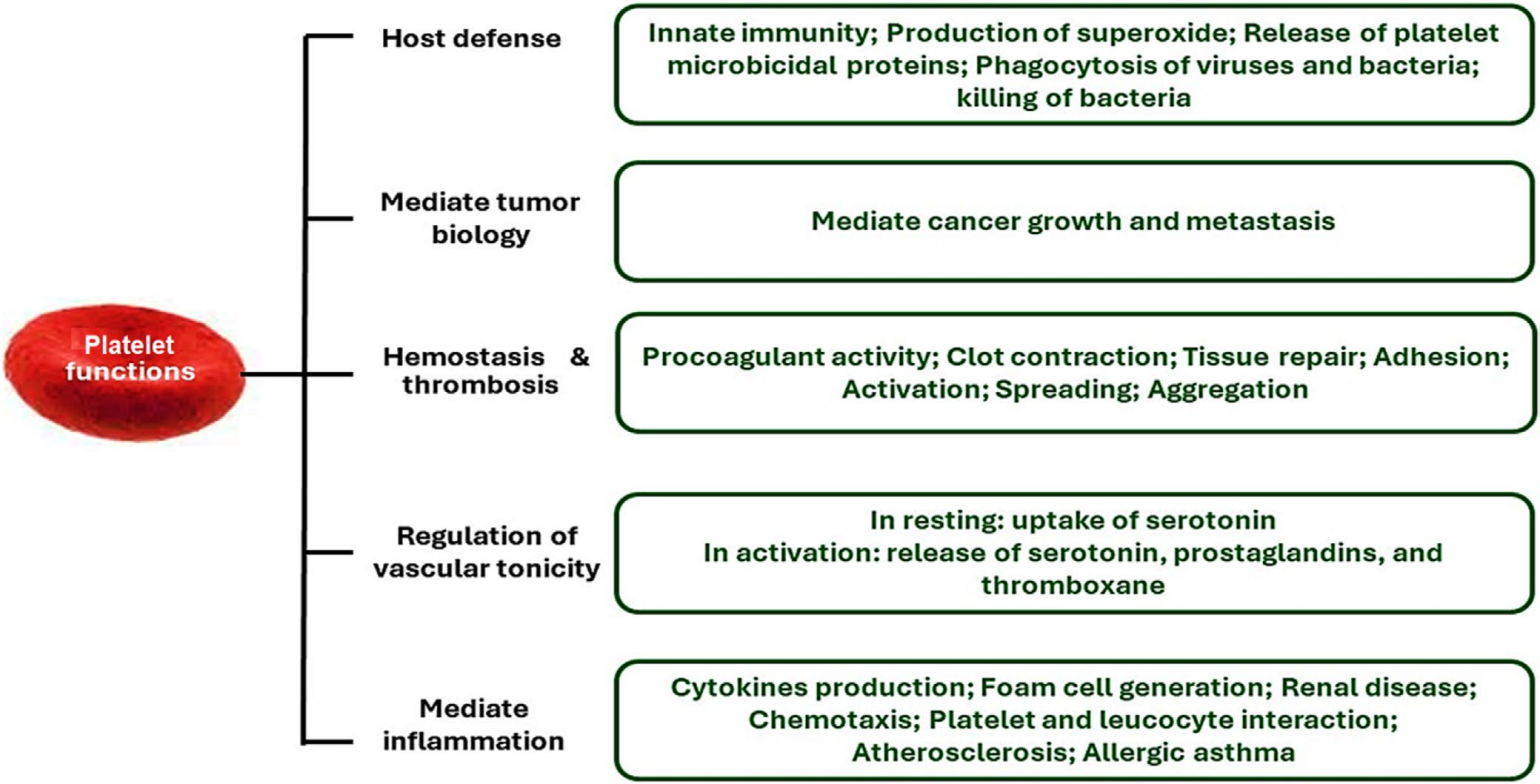
Hemostatic and non‐hemostatic functions of platelets: See the text for details.

In addition, platelets produce numerous lipid mediators such as thromboxane A2/TXA2 and platelet‐activating factor (PAF) [26]. Thromboxane A2 is released from activated platelets involved in the induction of platelet aggregation [28]. PAF is a signaling molecule expressed on the platelet surface membrane that is also produced from neutrophils, endothelial cells and neurons, and is involved in platelet activation [29]. In addition, PAF acts as a chemo‐attractant to immune cells, mainly monocytes, is involved in the differentiation of monocyte to macrophages, and increases the permeability of endothelial cells [29]. Of note, α‐granules contain PF4 (platelet factor 4), VWF (von Willebrand factor), SELP (selectin P), complement components, and CD40LG (CD40 ligand) [30]. Therefore, platelets are intricate in hemostatic and non‐hemostatic functions (Figure 2).

## 3 The Biological Function of APP

**FIGURE 3:**
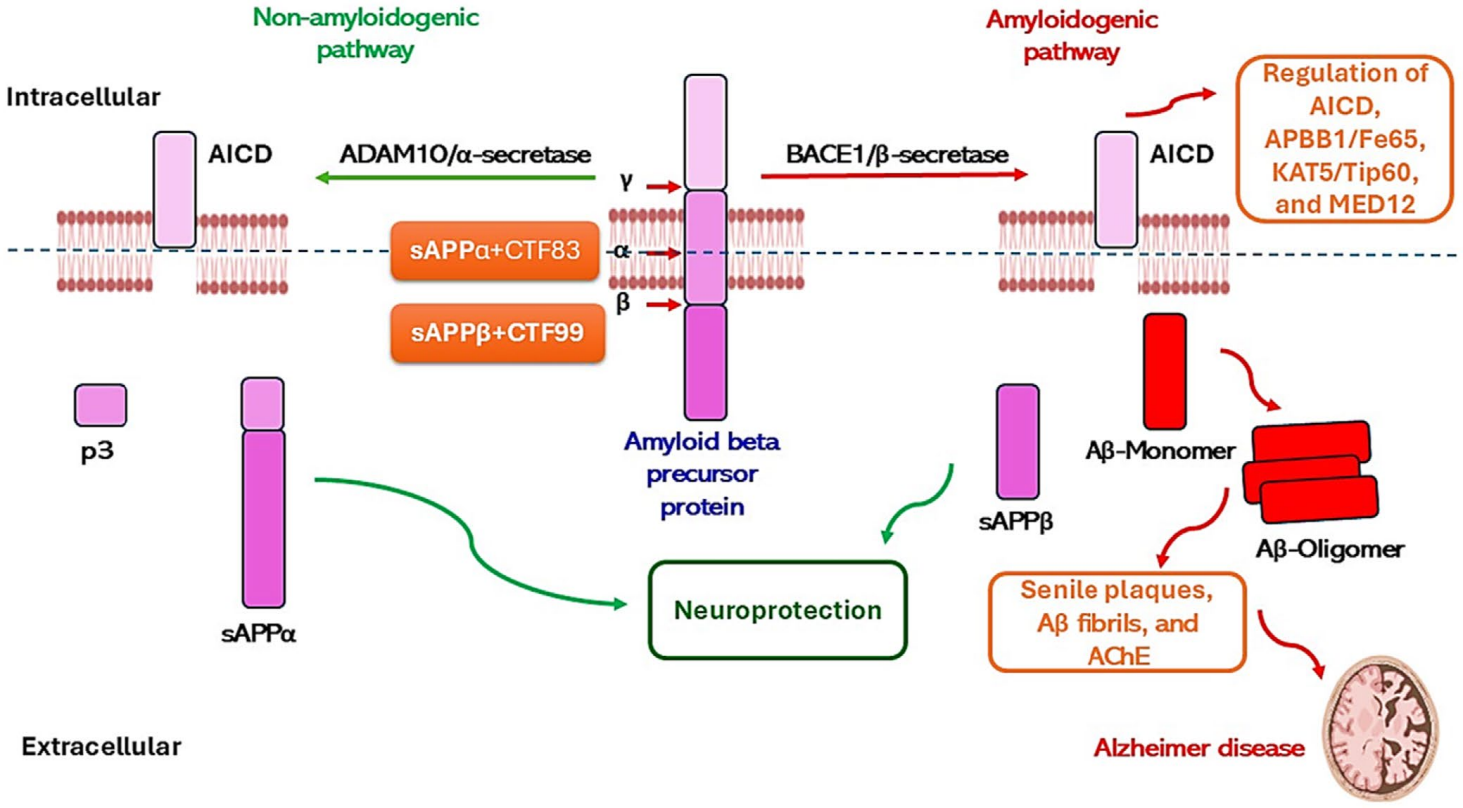
Processing of APP. Cleavage of APP by ADAM10/α‐secretase or BACE1 generates a soluble N‐terminal fragment (sAPPa or sAPPβ). The C‐terminal fragments (CTFs) from α‐secretase or BACE1 cleavage of APP are referred to as CTF83/C83 and CTF99/C99. γ‐secretase cleavage of CTF83 generates p3, whereas cleavage of CTF99 produces Aβ; this cleavage also produces the N‐terminal APP intracellular domain (AICD). Thus, cleavage of APP by BACE1 and γ‐secretase generates Aβ, and cleavage of APP by ADAM10/α‐secretase prevents Aβ formation. AICD can bind APBB1/Fe65 (amyloid beta precursor protein binding family B member 1), allowing recruitment of KAT5/Tip60 (lysine acetyltransferase 5), subsequent translocation into the nucleus and transcriptional activation of various target genes. MED12 (mediator complex subunit 12) is another AICD‐binding protein that acts as a transcriptional coactivator.

The glycoprotein APP and its related proteolytic fragments have different biological functions. APP may serve as a membrane surface receptor as many proteins can interact with APP [31, 32]. APP interacts with different adhesion molecules, which affects the balance of its processing in amyloidogenic versus non‐amyloidogenic pathways [32] (Figure 3).

APP facilitates the adhesion of Aβ to the neuronal membrane and promotes Aβ‐induced neurotoxicity [32]. APP can interact with NOTCH protein signaling in the regulation of neurogenesis and gliogenesis [33]. Of interest, the signaling molecule SPON1/F‐spondin (spondin 1) may represent a ligand for APP that affects neuronal differentiation [34]. SPON1 inhibits APP processing by suppressing BACE1 activity [34]. Furthermore, APP can bind surface proteoglycans such as ITG (integrin) and COL (collagen) [35]. In addition, APP through interaction with APBB1/Fe65 improves synaptic plasticity and synaptogenesis (Figure 3) [36]. Through the transcriptional regulation of a range of target genes, APP regulates the neuronal homeostasis of iron, copper, and zinc [37]. Moreover, APP can modulate insulin release and glucose metabolism in mice [38, 39]. In addition, APP regulates neuronal cholesterol metabolism [40]. In particular, the CTF99 fragment of APP increases internalization of cholesterol in an AD mouse model [40]. These findings indicate that APP has different physiological roles in neuronal metabolism. However, some mutant forms of APP increase the production of Aβ through the amyloidogenic pathway, leading to the development of AD neuropathology.

Platelets contain comparable amount of APP to that present in the brain [41]. Platelets contain two major APP isoforms (APP751 and APP770) [42]. Approximately 90% of platelet APP is soluble compared to 50% of neuronal APP [20]. Platelet APP prevents the activation of clotting factors F11/XI (coagulation factor XI) and F9 (coagulation factor IX) [21]. In addition, platelet APP has a procoagulant effect to maintain hemostasis, and lack of APP in mice increases the risk of bleeding [43]. Moreover, platelet APP has a moderate inhibitory effect on F7 (coagulation factor VII) and the F7‐F3 (coagulation factor III, tissue factor) complex [21]. In addition, platelet sAPPα inhibits clotting factors F11 and F9, thereby preventing thrombosis and preserving hemostasis [44]. Exogenous sAPPα inhibits platelet aggregation [45]; however, the antiplatelet effect of sAPPα is inhibited by Aβ through an ADP‐dependent mechanism [45]. Platelet aggregation is augmented in an AD mouse model due to Aβ‐induced mitochondrial dysfunction of platelets, which induce aggregation of Aβ [45]. Therefore, APP has an important physiological function to preserve the homeostasis of neurons and platelets.

## 4 Alteration of Platelet APP Processing in AD

Many changes have been shown in platelets of AD patients compared to healthy controls. For example, APP processing is augmented toward the amyloidogenic pathway in platelets derived from AD patients [46]. In addition, MAOB (monoamine oxidase B), which is expressed in neurons for the metabolism of neurotransmitters, is also present in platelets. The expression of MAOB is increased in the brain and platelets of AD patients [47]. Likewise, ITG expression on the platelet surface is increased in AD patients [48]. Furthermore, coated platelets, which have higher pro‐coagulant activities and express APP, are increased in early AD patients [48].

Platelets have all of the enzymatic machinery necessary for the processing of APP [49], and platelets are the major source of peripheral APP and Aβ [50]. The expression of platelet APP is increased by 2‐fold in AD patients compared to healthy controls [51]. Alteration of the expression of platelet APP is correlated with a reduction of ADAM10/α‐secretase and an increase of BACE1 [51]. In addition, platelet APP processing is increased with the generation of different APP fragments including Aβ in AD patients [20]. Platelets can produce all APP fragments such as Aβ, CTF99, sAPPα, and sAPPβ [52]. Unlike neuronal APP, which is processed by BACE1, platelet APP processing is mainly through the non‐amyloidogenic pathway by ADAM10/α‐secretase in a CALM (calmodulin)‐dependent manner [49]. Therefore, the level of platelet sAPPα in the granules is more than Aβ [45]. However, the platelet sAPPα level is reduced in AD patients [51, 53, 54]. The activity of ADAM10/α‐secretase in the platelets derived from AD patients is decreased by 50% compared to healthy controls [53]. Furthermore, the cerebrospinal fluid sAPPα level is low in AD patients compared to healthy controls [53]. A case control study found that the activity of ADAM10/α‐secretase is reduced in sporadic AD patients compared to healthy controls [54]. In addition, the activity of platelet BACE1 expression is increased by 17% in sporadic AD patients compared to healthy controls [55]. However, platelet BACE1 is not correlated with cognitive impairment in AD patients [55], suggesting that platelet BACE1 activity may precede AD neuropathology. Another study found that platelet BACE1 expression is increased by 24% in patients with mild cognitive impairment (MCI) compared to healthy controls [56] suggesting that platelet BACE1 is augmented in early AD. Conversely, both BACE1 and ADAM10/α‐secretase are observed as being unchanged in AD patients or patients with MCI [57]. However, the small sample size of this study and the reported variability in the measurement of BACE1 and ADAM10/α‐secretase may limit its significance.

In addition, platelets produce less aggregating Aβ40 compared to neurons, which produce aggregating Aβ42, and most of the circulating Aβ is derived from activated platelets [57]. Activation of platelets triggers the release of only 30% of stored Aβ40 [50]. Of interest, exaggeration of platelet activation is correlated with AD neuropathology through the development of cerebral amyloid angiopathy [58]. Consistently, activated platelets are increased in amnestic patients with MCI compared to non‐amnestic patients with MCI [59]. Furthermore, activated platelets are correlated with cognitive impairment in AD patients [60]. The biomarkers of platelet activation such as SELP and ITGA2B/glycoprotein IIb‐ITGB3/glycoprotein IIIa are increased in AD patients with faster cognitive decline compared with those with slow cognitive decline [60]. These findings highlight the conclusion that platelet activation and associated changes in the processing of APP are linked with the pathogenesis of AD.

## 5 The Link Between Activated Platelets and AD Neuropathology

Mounting evidence highlights a close relationship between activated platelets and the risk of AD. A cohort longitudinal study illustrated that subjects free of antiplatelet treatment followed‐up for 20 years are at higher risk for the development of AD [61]. Many studies indicate that activated platelets promote the conversion of soluble Aβ to the neurotoxic amyloid plaques [62, 63]. A preclinical study suggested that AD increases the risk of atherosclerosis through activation of peripheral platelets [62]. Kniewallner and coworkers found that platelets in a transgenic AD mouse model are activated and can cross the blood–brain barrier (BBB) to reach the brain and activate astrocytes [64]. Invasion of the BBB and brain parenchyma by activated platelets is mediated by the release of metallopeptidase [65]. Activated platelets in the brain release inflammatory mediators and return to the circulation, or are eliminated by microglia [66]. However, platelet‐astrocyte interaction prolongs the residence of activated platelets in the brain, and through the release of inflammatory mediators leads to neuroinflammation, demyelination, and neurodegeneration [67]. Interestingly, platelets in brain parenchyma become fully activated in response to AD neuropathology [64]. In addition, activated platelets in AD have structural changes due to alterations in the platelet contents that occur in response to oxidative stress and inflammation [64]. These findings indicate mutual interactions between activated platelets and AD neuropathology. Hence, peripheral platelet biomarkers could be used as diagnostic and prognostic tools [68].

Vascular injury and dysfunction are involved in the development and progression of neurodegeneration in sporadic AD [69]. The underlying cause for vascular injury in AD is related to Aβ‐induced platelet activation and damage of the vascular endothelium [69]. Aβ from brain amyloid can pass through the BBB to the cerebrospinal fluid and reach the cerebral vasculature leading to cerebral vascular angiopathy [70]. Augmentation of vascular Aβ and reduction of perivascular clearance of Aβ contribute in the pathogenesis of cerebral vascular angiopathy and AD [70]. Furthermore, Aβ can activate platelets through activation of platelet GPIBA/GPIbα (glycoprotein Ib platelet subunit alpha) and CD36 (CD36 molecule (CD36 blood group)) receptors with subsequent stimulation of PTGS/COX (prostaglandin‐endoperoxide synthase), thromboxane A2, and MAPK (mitogen‐activated protein kinase) [71]. In addition, Aβ‐induced inflammation and oxidative stress increase platelet activation [71]. Beura et al. [72] proposed that oxidative stress‐induced neuroinflammation and neurodegeneration in AD are involved in the activation of peripheral platelets.

Furthermore, activated platelets contribute to the pathogenesis of AD. Evidence from a preclinical study confirmed that the brain Aβ level correlates with the peripheral Aβ level [73] suggesting that activated platelets are implicated in the pathogenesis of AD. It has been proposed that activated platelets can release Aβ into the circulation that transports across the injured BBB to the brain leading to the formation of amyloid plaques [52]. Activated platelets also release other proteins such as SNCA (synuclein alpha), which is involved in the pathogenesis of Parkinson disease and AD variants [19]. Wu et al. [74] established that activated platelets can destroy the BBB and transport Aβ into the brain in an AD mouse model. Injection of platelets from an aged transgenic AD mouse to the brain of young mice accelerates BBB injury and augments the transport of peripheral Aβ into the brain. Aβ content in the platelets increases with age and contributes to neurodegeneration and AD neuropathology in aging [74].

Based on the above, targeting peripheral platelets by aspirin, which inhibits platelet activation by inhibiting PTGS/COX, may reduce AD risk. Along these lines, a population study illustrated that long‐term use of aspirin decreases AD risk in diabetic patients [75]. Aspirin inhibits the release of Aβ from activated platelets by blocking COL‐ and F2 (coagulation factor II, thrombin)‐induced exosome release [76]. Aspirin also inhibits Aβ aggregation in the brain preventing synaptic dysfunction and progression of AD neuropathology through a PTGS/COX‐dependent mechanism [77]. Aspirin, through modulation of cAMP‐PRKA/PKA (protein kinase cAMP‐activated) inhibits platelet activation and reduces Aβ release to the plasma [74]. Moreover, the platelet inhibitor clopidogrel has a neuroprotective effect against the pathogenesis of AD by reducing neuroinflammation and the accumulation of Aβ in rat hippocampus [78]. In addition, clopidogrel increases the acetylcholine level in the hippocampus comparable to the ACHE (acetylcholinesterase (Yt blood group)) inhibitor donepezil [78]. Findings from a preclinical study demonstrated that clopidogrel through inhibition of platelet P2RY12/P2Y12 (purinergic receptor P2Y12) attenuates Aβ aggregation in activated platelets and in the neurons of an AD mouse model. In addition, clopidogrel reduces the release of Aβ from activated platelets thereby reducing the risk of cerebral amyloid angiopathy and AD [79]. In addition, the released Aβ40 from activated platelets induces further platelet activation through activation of FG (fibrinogen), ITG, and GP6 (glycoprotein VI platelet) receptors [80]. Clopidogrel, through inhibition of FG receptors, inhibits the interaction between Aβ40 and platelets [81]. Therefore, clopidogrel may be effective against the development of cerebral amyloid angiopathy and AD by reducing the accumulation of Aβ in cerebral vasculature and brain neurons, respectively [20]. However, prolonged use of antiplatelet drugs may increase the risk of intracranial hemorrhage [82].

Interestingly, traditional therapeutic targets for inhibiting platelet activation have primarily been limited to PTGS1/COX‐1, integrin ITGA2B/*α*_IIb_‐ITGB3/*β*_3_, and P2RY12. Platelets become hyperactive in cases of obesity, diabetes, and hypertension, and in smokers. Upon activation, platelets can attract leukocytes and progenitor cells to vascular sites [83]. Platelets release various angiogenic factors promoting pathological reactions that also maintain sustained activation, further affecting these disease processes. Although the mechanisms are unknown, multiple stimuli induce platelet hyperreactivity but involve the early pathways of platelet activation. The exact mechanisms of how hyperactive platelets contribute to these diseases are still unclear, and antiplatelet strategies are inevitable for preventing these diseases. Reducing platelet function during the early stages could significantly affect these diseases [83]. Therefore, appropriate management of cardiometabolic risk factors such as obesity and hypertension can prevent platelet activation and reduce the risk of AD.

Furthermore, the expression of ADORA2A (adenosine A2a receptor) is increased in AD patients both in the hippocampus and peripheral platelets [84]. Normally, ADORA2A, which is expressed in neurons and synapses, regulates memory and cognition [85]. Overexpression of ADORA2A is linked with the activation of astrocytes and neurodegeneration in AD [86]. However, ADORA2A agonists have neuroprotective effects by inhibiting neurotoxicity, excitotoxicity, and neuronal apoptosis [87]. Therefore, modulation of ADORA2A in both platelets and neurons may reduce AD risk because downregulation of neuronal ADORA2A is also implicated in the pathogenesis of AD [88]. ADORA2A agonists have antiplatelet effects, and can overcome the resistance to the platelet P2RY12 antagonists such as clopidogrel [89]. Of note, adenosine has inhibitory effects on the central nervous system and stimulatory effects peripherally. Therefore, ADORA2A antagonists such as caffeine could be effective in the management of AD [90].

Of interest, anti‐AD medications such as tacrine and donepezil improve cognitive impairment and memory deficit but do not reverse the underlying AD neuropathology [91]. However, antiplatelet drugs, by inhibiting the release and processing of APP from overactivated platelets in AD patients, could be a potential preventative measure against the progression of AD neuropathology. Therefore, antiplatelet drugs in combination with anti‐AD medications might be a promising therapeutic strategy in the management of AD.

Taken together, activated platelets and linked APP alteration are highly implicated in the pathogenesis of AD. However, activated platelets in AD may be primary or secondary events, and this topic thus needs further research. Nevertheless, inhibition of platelets can reduce the peripheral amyloid burden and associated AD risk. The present review has many limitations including the fact that platelet activity was not estimated in different stages of AD, and in relation to anti‐AD treatments. Nonetheless, this review suggests a bidirectional relationship between activated platelets and AD neuropathology, and cutting this circuit loop may prevent the development and progression of AD.

## 6 Conclusions

AD is the most common neurodegenerative disease linked with the development of dementia. The hallmarks of AD neuropathology are the depositions of Aβ and NFTs. Aβ is derived from the processing of APP by BACE1 and γ‐secretase through an amyloidogenic pathway. Conversely, processing of APP by ADAM10/α‐secretase through a non‐amyloidogenic pathway produces the neuroprotective sAPPα. Notably, platelets have all of the enzymatic machinery necessary for the processing of APP and are regarded as the major source of peripheral APP and Aβ. Platelets can produce all of the APP fragments such as Aβ, CTF99, sAPPα, and sAPPβ. Unlike neuronal APP, which is processed by BACE1, platelet APP processing is mainly through the non‐amyloidogenic pathway by ADAM10/α‐secretase. The expression of platelet APP is increased by 2‐fold in AD patients. Alteration of the expression of platelet APP is correlated with a reduction of ADAM10/α‐secretase and an increase of BACE1. In addition, platelet APP processing is increased with the generation of different APP fragments including Aβ in AD patients. APP processing is augmented toward the amyloidogenic pathway in platelets derived from AD patients. Thus, activated platelets are implicated in the pathogenesis of AD, which also increases platelet activation. Therefore, there is a close relationship between activated platelets and AD neuropathology. Collectively, activated platelets and the linked APP alteration are strongly implicated in the pathogenesis of AD. However, activated platelets in AD may be primary or secondary events. Nevertheless, inhibition of platelets can reduce the peripheral amyloid burden and associated AD risk. Additional preclinical and clinical studies are warranted in this regard.

## Author Contributions

Conceptualization, Hayder M. Al‐kuraishy, Ghassan M. Sulaiman, Hamdoon A. Mohammed, and Daniel J. Klionsky; Software, Hamdoon A. Mohammed, and Ali K. Albuhadily; Writing – original draft preparation, Hayder M. Al‐kuraishy, Ghassan M. Sulaiman, Ali K. Albuhadily, and Ali I. Al‐Gareeb; Writing – review and editing, Hamdoon A. Mohammed, Retaj A. Dawood, Daniel J. Klionsky, Ali I. Al‐Gareeb, and Mosleh M. Abomughaid; Supervision, Hayder M. Al‐kuraishy, Ghassan M. Sulaiman, and Daniel J. Klionsky; Project administration, Ghassan M. Sulaiman and Daniel J. Klionsky. All authors have read and agreed to the published version of the manuscript.

## Ethics Statement

The authors have nothing to report.

## Conflicts of Interest

The authors declare no conflicts of interest.

## Data Availability

The authors have nothing to report.
